# Osmosis

**DOI:** 10.5195/jmla.2020.949

**Published:** 2020-04-01

**Authors:** Christy Tyson

**Affiliations:** Manager, Library Services, Ascension Health, Nashville, TN, Christina.tyson@ascension.org

## INTRODUCTION

**Figure 1:**
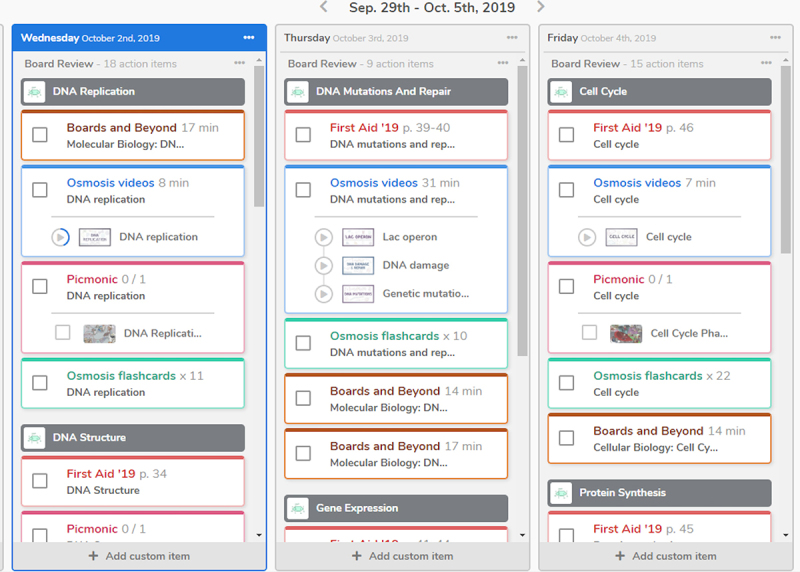
Screenshot of mock calendar created to track and organize a graduating medical student who is studying for board exams, with a timeline of October through May Used with permission; 2 October, 2019; Osmosis.

Osmosis was developed and founded by Shiv Gaglani and Ryan Haynes, while they were attending medical school at Johns Hopkins. Aware of how much time they were spending on preparing their study resources rather than learning medicine, Gaglani and Haynes created a personalized learning platform that is competency based and scaffolds content using algorithms that consider information generated by student use. Scaffolding is the use of multiple teaching strategies to reinforce content, moving a student toward mastery. Use of educational technologies and the strategy of scaffolding has been linked to more successful disciplinary decision making [1]. Osmosis allows students to schedule how, when, and in what format they will access content to study (Figure 1). They can then integrate social media sites to communicate with other medical and health sciences program learners.

Over the last two decades, medical education has moved toward a competency-based model, paralleling changes in the type of learning that millennials prefer. Often referred to as “digital natives,” millennials incorporate technology and social collaborative aspects of technology into the entirety of their world [2]. Osmosis speaks to the millennial user by utilizing contemporary terms such as “playlists,” a term used on platforms such as Spotify and Google Music to refer to lists of songs. Osmosis users can organize study content into playlists, arranging them to fit their personal study habits and preferences, thus using educational technologies to make learning more engaging.

## CONTENT

Osmosis does not limit its use to students. The development team also provides personalization from an institutional curriculum perspective. Faculty reviewers (both inside and outside Osmosis) provide learning objectives and content that is then supported and extended through interactive media. Osmosis maps to learning objectives for current curricula by discipline, topic, and certification-related content. All material is presented at levels that are easy to follow with logical sequencing creating the bridges needed between topics for students to achieve success.

Videos that combine medical illustration, radiographs, computed tomography (CT) scans, and more allow students to connect written-word to real-world applications. In this reviewer’s opinion, Osmosis incorporates learning theory and curriculum development in its platform. Osmosis has certified curriculum designers who collaborate with all stakeholders to verify that content is presented through appropriate venues for adult learners. Additional evidence of Osmosis’s commitment to best practice for evidence-based learning is through a permanent link on the user dashboard. When one clicks on “Proven learning methods,” a series of videos—“How to Succeed in Health Professions”—illustrates these points and explains the Osmosis methods for:

- retention techniques
- learning
- teaching
- career development and other resources

Integrated content is continually reviewed for updates and accuracy. Additions and updates are announced to subscribers through the dashboard feature in the platform and by email so that students and faculty can return to these topics for review.

**Figure 2:**
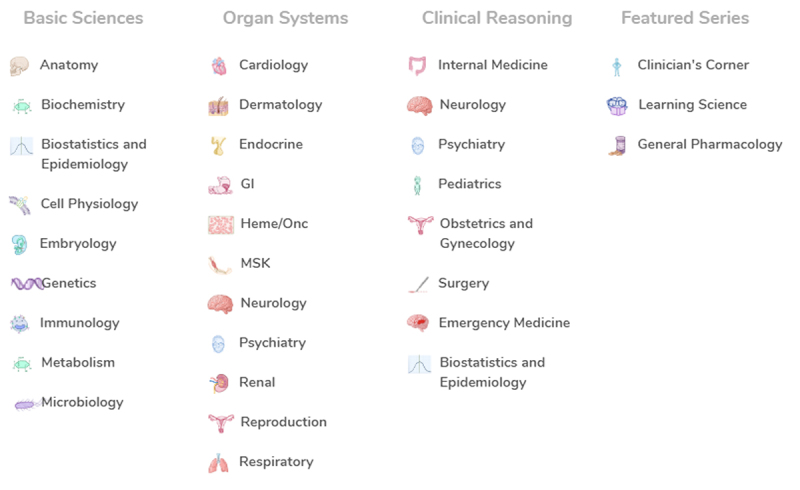
Screen shot of Categories with subjects underneath Used with permission; 2 October, 2019; Osmosis.

A wide range of topics are covered under the following categories: Basic Sciences, Organ Systems, Clinical Reasoning, and Featured Series (Figure 2).

## LIMITATIONS

Osmosis is primarily focused on medicine, but additional content was rolled out in July 2019. Content specific to study for registered nurse, nurse practitioner, physician assistant, dentistry, and other allied health professions that is based on content standards and evidence-based education techniques is now included. Currently, these newer additions are not as robust as medicine and its specialties; however, Osmosis representatives indicate that this content will continue to grow over time.

## MAJOR FEATURES

Osmosis’s interactive study scheduler is the key feature in keeping users’ content organized across a personalized time line. Additional features effectively plug content into the scheduler using a variety of presentation methods. These features include customizable playlists of media content, flash cards, and question banks. All features track content that is accessed and user responses, and then create and deliver new lists, flash cards, and question banks that are personalized to each user via the dashboard. It is the platform’s advanced series of algorithms that allow Osmosis to continually present personalized remediation, thus taking the burden of tracking content review needs from the user.

Video content takes the form of Picmonic in Osmosis. Picmonic utilizes visual learning and gaming theory to test understanding. This reviewer found that Picmonic provides an enjoyable and relatable way to uptake and assess content retention. Osmosis also tracks videos that are watched and integrates these choices into review and question banks.

## AUDIENCE AND PURPOSE

Osmosis is an online learning platform that offers educational content for medical students, nursing students, and health care professionals. Certification resources are available in first aid as well as board preparation for medical students. Board preparation options take into consideration discipline, specialties, and subspecialties directed toward specific content needed for exams such as United States Medical Licensing Examination (USMLE) step 1 and step 2.

## ACCESSIBILITY AND USABILITY

Osmosis is supported in Google Chrome, Firefox, Safari, and Opera. It does not function in Internet Explorer. The platform is very user friendly, with simple but effective icons and descriptions for buttons and links to content. A mobile app is available for iOS and Android devices, providing study on the go as well as allowing listening to content while offline. Students can link to Pathoma, SketchyMedical, and Boards&Beyond so that Osmosis can integrate all the content and variations on presentation that these other platforms offer.

From an instructional perspective and with site subscription, all Osmosis content is available to clip, annotate, and embed in learning management systems, PowerPoint, and other presentations, when cited correctly. Teaching faculty can front-load content, annotate in a didactic environment, and post in the learning management system for students to access later.

Subscriptions for single users are available at very reasonable prices and can be accessed at Osmosis for Medical Students (MD); institutional pricing is available upon request.

## BRIEF COMPARISONS

Although there are several platforms that offer similar content for study purposes, none offer the personalization and many of the features found in Osmosis.

GibLib utilizes 360-degree virtual reality, 4K and high definition (HD) videos of surgical procedures narrated by medical professionals to present surgery-specific content. GibLib has an app version, so the student or specialist can listen to narration without viewing procedures. A 4-day free trial is available. Pricing was not available at the time of this review.

Visible Body is focused on anatomy and physiology, and integrates some gaming features. Gaming controllers can be used to move within the platform and cut away body system layers. Visible Body has a higher price point than Osmosis and requires the purchase of hardware, such as the controllers, and extensive memory capabilities in gaming-specific hardware for the platform to function.

Finally, Firecracker is a digital tutoring system that covers fourteen of the major content areas of the Physician Assistant National Certifying Exam (PANCE), USMLE-style questions, and content of the Comprehensive Osteopathic Medical Licensing Examination (COMLEX). While this platform is most like Osmosis, it is not geared toward as many disciplines and does not integrate social media study platforms. There is, however, a clerkship support section.

## CONCLUSION

Osmosis is a strong choice for health sciences students who are looking for personalized study for board and program. The company partners with institutions to further personalize the online platform’s algorithms, integrating curricula and professional standards into students’ overall study plans. Extensive features include an adaptable study scheduler that embeds evidence-based, discipline, and topic peer-reviewed information that is appropriate for medical, dental, and nursing candidates. Tracts are also available for nurse practitioner and physician assistant students. Affordable individual and institutional based subscriptions are available, and certified curriculum specialists are on staff to further integrate institutional curricula and professional standards in the platform for subscribers and partners.

## 

***Christy Tyson, MLIS****, Christina.tyson@ascension.org, Manager, Library Services, Ascension Health, Nashville, TN*
